# Fetal Environment and Schizophrenia

**DOI:** 10.1289/ehp.7572

**Published:** 2005-05-26

**Authors:** Mark G. A. Opler, Ezra S. Susser

**Affiliations:** 1 Department of Psychiatry, Columbia University, New York, New York, USA; 2 Department of Epidemiology, Mailman School of Public Health, Columbia University, New York, New York, USA; 3 New York State Psychiatric Institute, New York, New York, USA

**Keywords:** developmental, lead, Pb, prenatal, prospective, psychosis, schizophrenia

## Abstract

Schizophrenia and related disorders are adult-onset illnesses with no definitively established risk factors. Several studies report that exposures to infection and nutritional deprivation during early development may elevate the risk of later developing schizophrenia, specifically during the prenatal period. Preliminary evidence implicates lead exposure as well, suggesting that chemical exposures during early development may constitute a new class of risk factors for schizophrenia that has not been adequately investigated. Exposure to lead is given as an example of a chemical agent for which some effects have been described throughout the life course on both general neurodevelopmental outcomes and now on a specific psychiatric diagnosis. Findings from prospectively collected birth cohorts are offered as examples of both innovations in methodology and opportunities for future generations of investigators.

Schizophrenia and schizophrenia spectrum disorders (SSDs) are mental illnesses of unknown etiology, typically diagnosed in adolescence or adulthood. With no known cure, these diseases are frequently associated with long-term disability and staggering social and economic costs. Over the past 20 years, researchers have theorized that exposures that elevate the risk of later developing schizophrenia may occur during the prenatal period. A current version of the “neurodevelopmental hypothesis” of schizophrenia states that gene–environment interactions alter the structure and function of the developing brain, contributing to the onset of schizophrenia later in life (Murray and Lewis 1987; Waddington and Youssef 1987; Weinberger 1987). Although this working hypothesis is now widely used, the underlying mechanisms are the subject of ongoing debate. In this review we consider methods being used to study the prenatal environment and schizophrenia, particularly the relationship between prenatal lead (Pb) exposure and schizophrenia.

## Early Antecedents of Schizophrenia

Schizophrenia is a mental illness that has been grouped among the psychotic disorders, that is, those chiefly characterized by delusions, sensory hallucinations, and severe impairments of speech organization. It has not been associated successfully with any specific physical finding universal to all cases. For some time, this led researchers to doubt that schizophrenia had any underlying physical cause. Many sought to find evidence of brain pathology but were unsuccessful, leading to the declaration that schizophrenia was the “graveyard of neuropathologists” (Plum 1972). During 1980s, that belief was gradually supplanted as subtle physical findings began to emerge. Neuroimaging techniques suggested structural changes, such as increased ventricular volume in the brains of patients (Kelsoe et al. 1988), and cohort studies found differences in neuromotor function and childhood behavior between patients who developed schizophrenia and the general population. This seemed to suggest early origins of the illness, predating clinically defined disease by decades. Although on brain imaging, certain regional changes such as increased ventricular and reduced cortical and hippocampal volume have been noted (Halliday 2001), findings in schizophrenia are comparatively subtle. Overall, these indicate mild structural disarray at the cellular level, changes in neural density (Chana et al. 2003), and decreased neuropil (Roberts et al. 1996) along with altered connectivity in multiple regions of the brain (Harrison 1999).

### Prospective approaches.

The use of postmortem examination is problematic in studies of diseases that have early antecedents because it is difficult to determine whether findings are because of underlying dysfunction rather than degeneration after clinical progression or side effects of pharmacologic treatment. In the study of fetal origins of schizophrenia, it is also difficult to apply the case–control approach, because reliable evidence of events that occurred during fetal development cannot be obtained easily. Prospective techniques are one potential solution; such studies, based on cohorts of subjects identified before the onset of disease and before the exposures under investigation (e.g., at or before birth) and followed across the life course, have become key to investigations of early developmental events in schizophrenia. However, such investigations are complicated by the very long latency of the disease, since clinical onset may not be evident until adolescence or adulthood, decades after the putative prenatal exposures. Systems to track subjects, identify exposures, and diagnose disease must be maintained for decades. In addition, large numbers of subjects are required to accurately assess the relatively modest increases in risk that any single factor is likely to contribute to a multifactorial disease such as schizophrenia.

Recent investigations have built on these studies, using prospective cohorts identified before birth for studies of known or suspected neurodevelopmental disruptors. Several ascertain prenatal exposure through quantifiable measurements, for example, analysis of archived maternal biologic samples collected before birth. Various hypotheses have been advanced, and a number of studies have produced suggestive results. As an example, we describe one ongoing study that has examined toxic, nutritional, infectious, and other risk factors. After describing selected findings on infection and nutrition that illustrate the methods used, we then describe how this study has been used to investigate prenatal lead exposure as a risk factor.

### The PDS study.

The Prenatal Determinants of Schizophrenia (PDS) study was initiated in the 1990s. It is based on a cohort of approximately 20,000 pregnant women identified in northern California between 1959 and 1966 as part of the Childhood Health and Development Study (Susser et al. 2000). This study includes aliquots of maternal sera drawn during prenatal visits. These samples were stored and maintained at National Institutes of Health facilities, frozen at −20°C in anticipation of future studies. They have been used in combination with hospital records and new diagnostic data (van den Berg et al. 1988).

Cases of schizophrenia and SSDs were identified from a database of inpatient, outpatient, and pharmacy records. Records for cohort members with diagnoses indicative of psychosis or prescriptions for antipsychotic medication were reviewed, abstracted, and rated by two psychiatrists for the presence or absence of psychosis. These ratings were then used to identify potential cases to be sought for a thorough diagnostic interview. Ultimately, 71 cases of schizophrenia and SSDs were identified. Controls were selected from the cohort and matched to cases on the basis of several factors, including date of membership in the cohort, date of birth, gender, timing of first maternal blood draw, and the number of available serum samples (for details see Susser et al. 2000).

### Influenza and markers of infection.

Previous work describing associations between prenatal exposure to a variety of viral agents has been considered for some time and extensively reviewed elsewhere (Crow 1978; Mednick et al. 1988; Torrey and Peterson 1973). The PDS study is among the first studies capable of performing serologic measures for exposure to influenza. For this analysis in stored maternal serum from cases and matched controls, the hemagglutination inhibition test was performed on four antigens of influenza strains known to be prevalent between 1959 and 1966 in northern California, including A/H2N2/Japan/57, A/H2N2/Japan/62, A/H2N2/Taiwan/64, and B/Massachusetts/66. Exposure to influenza usually results in a rise in antibody titers, referred to as seroconversion. Typically, seroconversion is characterized as a 4-fold rise in antibody titers taken in serial samples. As most subjects in this study had single samples taken within each trimester, a single cutoff level was sought as a proxy of influenza exposure during pregnancy. Validity studies demonstrated that levels of ≥ 1:20 in a single serum sample were highly specific and sensitive.

First-trimester exposure was associated a 7-fold increase in risk of schizophrenia and SSDs, whereas second- and third-trimester exposure showed no increase in risk. However, although first trimester is usually defined as the period between zero and 90 days after the last menstrual period (post-LMP), the blood draws taken in this study only occur as early as 46 days post-LMP. Therefore, first trimester here signifies, in effect, assessment in the latter part of first trimester. Additional analyses were conducted analyzing exposure during the first and second halves of pregnancy defined as 0–142 days (in effect, 40–142 days post-LMP) and from 143 days post-LMP until termination of pregnancy, respectively. Exposure in the first half of pregnancy conferred a 3-fold increase in risk, whereas no increase was seen after exposure during the second half of pregnancy or when second-trimester exposure was considered.

Although clearly an advance over previous work, the PDS study has three key limitations. First, the number of cases of schizophrenia and SSDs with the required prenatal sera was small—64 cases and two matched controls per case. Although the study found a substantial association between prenatal influenza exposure and schizophrenia, the confidence limits of this association are wide. Second, influenza infection is typically documented by noting an increase in titers over time, and the measure used in this study represents a proxy of the established standard. Third, the increase in risk does not correspond exactly with previous findings concerning timing of exposure. Prior reports have indicated that second-trimester exposure is associated with increases in risk, whereas in this study, exposure during first trimester and first half of pregnancy confers risk. Further investigation is required to explain this difference.

### Prenatal maternal nutrition and body mass index.

Nutritional factors has also been postulated to play a role in the etiology of schizophrenia. Both lack of specific micronutrients and general nutritional deprivation have been previously implicated as risk factors for broad developmental disruption and for schizophrenia specifically. In one landmark study of prenatal nutritional deprivation known as the Dutch Famine Study (Susser et al. 1998), neurodevelopmental outcomes were measured after severe caloric restriction. Rates of schizophrenia approximately doubled for individuals conceived under conditions of nutrient deprivation during early gestation (Susser et al. 1996). Early gestational exposure to famine conferred risk for schizophrenia, whereas late gestational exposure did not. Later studies that extended these findings to schizophrenia spectrum personality disorders also showed a 2-fold increase in risk for early gestational exposure to famine (Hoek et al. 1998). Two other studies found evidence that low maternal body mass index (BMI) or low birth weight is associated with schizophrenia (Done et al. 1991; Wahlbeck et al. 2001).

Recently, high rather than low maternal BMI has become a focus of concern because the number of women of reproductive age with above-average or high BMI has increased in industrialized societies. The PDS study used measures of prepregnant maternal BMI, categorized to low (< 19.9), average (20–26.9), above average (27–29.9), and high (≥ 30.0). Compared with average maternal prepregnant BMI, high BMI was significantly associated with schizophrenia and SSDs in the adult offspring (relative risk = 2.9; 95% confidence interval, 1.3–6.6). This finding was independent of maternal age, parity, race, education, or cigarette smoking during pregnancy.

## Prenatal Lead Exposure and Neurodevelopment

For centuries lead has been known as a toxic agent but only recently has been recognized as having subtle but significant developmental effects. McKhann stated in 1926 that the “manifestations of Pb poisoning usually subside without serious consequences.” In 1943 Byers and Lord (1943) disproved this statement in a follow-up study of 20 children with documented Pb poisoning. They examined not only gross neurologic signs but also IQ scores and academic performance. Although based on a small sample of convenience, 19 of the children later exhibited serious difficulties in school. Since these initial studies, prenatal Pb exposure has been measured using maternal blood Pb (BPb) during pregnancy, neonatal BPb, amniotic fluid, and umbilical cord BPb (Korpela et al. 1986). Comparisons of maternal and umbilical BPb indicate that transfer of Pb from maternal to fetal blood during pregnancy is unimpeded by the placenta. Prospective approaches to Pb exposure and development have been used in a number of instances. They have focused primarily on developmental outcomes such as attention, academic achievement, and cognition, and have used maternal blood draws or postnatal measures in a variety of biologic media (Pocock et al. 1994). These studies and others generally have provided strong support for the role of Pb as a developmental neurotoxin (Bellinger et al. 1994). However, because they mostly have followed subjects into or through childhood, they are not informative regarding adult-onset disorders such as schizophrenia.

A few modestly sized studies have now followed subjects through adolescence. In one example, Needleman and colleagues recruited 312 first- and second-grade children in Chelsea and Somerville, Massachusetts. Dentin Pb levels were measured for each subject (Needleman et al. 1979). This measure was used to identify high exposure (those with dentin levels > 20 ppm), moderate exposure (10–19.9 ppm), and low exposure (< 10 ppm) (Needleman et al. 1990). Neurobehavioral testing was conducted at the time of collection in 1979 (mean age, 7.3 years) and again in 1988 (mean age, 18.4 years). Dentin Pb is useful as a measure of exposure averaged over the age of the tooth, although dentin Pb levels are associated with dental caries and fillings (Gil et al. 1996). Results showed an increased risk of not graduating from high school among those with increased dentin Pb levels. Reading difficulties sufficiently severe to be defined as a disability showed a similar distribution. Subjects who had been diagnosed with clinical Pb poisoning earlier in the study had the highest percentages of failure to graduate (42.9%) and reading disabilities (50%).

A study by Dietrich et al. (2001) presents data that show Pb exposure versus juvenile delinquency at different exposure levels. Based in Cincinnati, Ohio, the sample of 195 subjects is largely African American, disadvantaged, and urban. Using a prospective cohort with prenatal and postnatal BPb assessments collected every 3 months until 6.5 years, the study measures parental and self-report of delinquent behaviors including drug and alcohol use in adolescence. Subjects were given self-report questionnaires and assessed at 15 and 17 years of age. The results are categorized by lowest, low, medium, and highest BPb levels. When prenatal BPb, average childhood BPb, and 78-month BPb were estimated as predictors of delinquent behavior, increasing concentrations were associated with a modest increase in delinquent acts reported in adolescence. Prenatal BPb exposure > 10 μg/dL results in an increase of more than 2.3 delinquent acts compared with exposures ≤ 5 μg/dL. Significantly higher rates of delinquent behavior are related via a categorical BPb measured prenatally and at 78 months of age, although not by average childhood BPb (Dietrich et al. 2001).

## Findings on Prenatal Lead Exposure and Schizophrenia

Many of the effects described in adolescence after early-life exposure, including decreased academic attainment, social deficits, and behavioral difficulties are comparable with the early antecedents of SSDs. Similarly, a number of factors that have been suggested as being associated with Pb exposure, such as urban residence, have also been studied as risk factors for SSDs. Although the samples from prospective studies described here do not have sufficient power to be definitive, the findings are suggestive, and the overall approach that these studies take may be used as a model. Principally, the combination of prospective collection of biologic samples can be combined with longitudinal assessments for the study of early-life exposures as they relate to adolescent and adult psychiatric diseases.

Although several techniques are available for assessing Pb exposure in biologic samples, the principal one used in studies of prenatal exposure is direct measurements on maternal blood. The PDS study has stored sera, not whole blood, containing the Pb-sequestering erythrocytes required for direct measurements in small volumes. Techniques for direct measurement of Pb could not be employed. However, a biologic marker of Pb exposure, δ-aminolevulinic acid (δ-ALA) may be detected in urine, plasma, and serum using high-pressure liquid chromatography with fluorescence detection.

Feasibility studies were conducted to assess the utility and predictive value of this technique in small volumes of stored maternal serum (Opler et al. 2004). It was determined that second-trimester serum was likely to be the best indicator of prenatal exposure because both Pb and corresponding δ-ALA levels are believed to be relatively stable at midpregnancy. Second-trimester samples were available for 44 cases and 75 matched controls (one to two controls per case).

A single 100-μL aliquot of second trimester serum was made available for each subject. A concentration of 9.5 ng/mL of δ-ALA, corresponding to a BPb level of 15 μg/dL, was used as a cut-off value to divide the sample into exposed and unexposed subjects. Samples were coded and blinded with respect to case status. Using this approach, Pb exposure as measured by elevated δ-ALA was associated with about a 2-fold increase in risk of SSDs in this sample (odds ratio = 2.43; 95% confidence interval, 0.99–5.96). The small numbers of subjects contribute to the wide confidence limits.

Some important limitations should be noted. First, the use of a biologic marker rather than direct measurements means that the observed increase in risk could be mediated by the effects of Pb on δ-ALA rather than by Pb exposure directly. Serum δ-ALA itself may be the exposure of interest. In experimental models, δ-ALA has been shown to be neurotoxic, interfering with GABA (γ-aminobutyric acid) neurotransmission [Emanuelli et al. 2001; also reviewed by Cory-Slechta (1995)]. Second, the findings of this study are also difficult to interpret conclusively because the sample size is relatively small and the result has a wide confidence interval. We have now obtained permission to analyze the one other existing data set of this type with a similar sample size. These results will be forthcoming.

## Future Directions

The use of prospectively collected cohorts in combination with archived biologic samples is a proven and powerful method for studying disease–exposure relationships throughout the life course. This method has allowed schizophrenia research to move away from less refined definitions of prenatal exposure and into investigations that may someday focus on specific molecular agents in causal pathways. This longitudinal approach was made possible by the foresight of early generations of researchers in combination with the efforts of those who succeed them. The initial results from prenatal cohort studies are still preliminary, and the process they describe is still in its infancy. Every class of candidate exposures will benefit from continued technical and methodologic refinement. Using infection as an example, those agents or strains that cause the greatest increases in risk, specific physiologic responses to infection, and the timing of exposure during pregnancy may be further investigated. Nutritional deprivation might also be explored in greater detail, using methods to study the roles of individual micronutrients. Finally, chemical exposures could eventually be examined in terms of toxicokinetics and mechanisms of action, allowing proximal effects of exposure to be teased apart and considered separately from consequent physiologic responses.

Although life-course epidemiology is currently yielding important results, it is limited to the resolution and specificity that designers of prenatal cohorts create through the type, frequency, and periodicity of data collection. Presently, the biology that links exposures to causal mechanisms is nearly impossible to study in detail without the use of experimental models. Basic researchers have used clinical and neurochemical observations from humans to develop of animal models of schizophrenia and are now studying some of the exposures implicated in epidemiology using the same techniques. Although animal models of psychiatric disorders are imperfect and subject to a number of limitations, they are useful for testing the biologic plausibility of new hypotheses generated by epidemiology.

We believe that to reach the goal of effective prevention of schizophrenia, all available data on the disorder must be integrated, including observational and experimental findings. Investigators with interdisciplinary training and who are comfortable with the language and concepts of study design from the population level to the molecular level will play a crucial role in the future of the field.
